# Case report: A rare case of multifocal, combined lymphatic-venous malformation

**DOI:** 10.3389/fmed.2024.1453312

**Published:** 2024-10-21

**Authors:** Hien Duy Pham, Duy Le Dinh Tran, Thom Hoang Dang, Luan Vo Mac Nguyen, Trang Thu Dang, Linh Vu Thuy Nguyen, Quang Thanh Nguyen

**Affiliations:** ^1^Department of Pediatric Surgery, National Hospital of Pediatrics, Hanoi, Vietnam; ^2^College of Health Science, Vin University, Hanoi, Vietnam

**Keywords:** vascular malformation, lymphatic malformation, venous malformation, pediatric, case report

## Abstract

Vascular malformations, including lymphatic-venous malformations (LVMs), are complex and can lead to significant morbidity. This case report details the management of a rare multifocal, combined LVM in a 3-month-old male, misdiagnosed as a right inguinal hernia prenatally. Postnatal imaging revealed multiloculated cystic masses extending from the mons pubis to the right groin, scrotum, and thigh. Doppler ultrasound and MRI demonstrated the extent of the masses, with the largest cyst measuring 4 × 4 × 2 cm. Initial surgical excision and debulking were performed; however, the lesion recurred 6 months later. Given the complexity and involvement of truncal vessels, complete excision was not feasible, and sclerotherapy with intralesional Bleomycin injections was initiated. After the first Bleomycin injection, a significant reduction in lesion size was observed. However, 6 months later, the lesion recurred and enlarged, necessitating a second Bleomycin injection. Subsequent follow-ups showed further reduction in lesion size. Unfortunately, 2 years later, the patient experienced diffuse cystic infiltration of the pelvis and right lower extremity due to treatment delays caused by the COVID-19 pandemic. Imaging at this stage revealed a cystic mass in the right pelvic cavity measuring 10 × 6 × 5 cm. Two additional sessions of Bleomycin injections were performed, resulting in a successful resolution of the cystic masses. Regular follow-ups have shown that the LVMs remain under control, with no symptoms and no concerns from the family. This case underscores the complexities involved in diagnosing and managing multifocal, combined LVMs. It suggests that Bleomycin sclerotherapy can be a valuable, minimally invasive alternative to more extensive surgical procedures, particularly when critical structures are involved. The patient’s condition has been successfully managed with a combination of surgical and sclerotherapy interventions, ultimately resulting in symptomatic relief and cosmetic improvement.

## Introduction

Vascular malformations, affecting approximately 1–1.5% of the population, include a spectrum of developmental anomalies involving capillaries, arteries, veins, and lymphatics (1). These anomalies, comprising both tumors and malformations, can cause significant morbidity and mortality in children and adults (2). Venous malformations are the most common, accounting for about 50% of cases, followed by arterial and arteriovenous malformations (approximately 35%), lymphatic malformations (around 10%), and mixed combined malformations (about 5%) (1).

The complexity and heterogeneity of these lesions, along with confusion regarding their classification, often lead to the misdiagnosis of vascular anomalies (3). The use of the International Society for the Study of Vascular Anomalies (ISSVA) classification system has been strongly recommended to distinguish between vascular tumors and malformations, enabling appropriate therapeutic measures (4). According to the ISSVA, vascular malformations are classified based on blood flow characteristics and the types of vessels involved: low-flow (lymphatic, venous, capillary, or combination) and high-flow (arterial or arteriovenous) (3). A combination of malformations may occur in the same patient, often resulting in a more complex disease that is challenging to understand and treat. We report a rare case of multifocal, combined lymphatic-venous malformations managed with surgical excision and Bleomycin sclerotherapy.

## Case presentation

A 3-month-old male presented with right inguinal and scrotal swelling, initially diagnosed prenatally as a right inguinal hernia at 8 months of pregnancy. Conservative management after birth was unsuccessful as the swelling increased with discoloration of skin, and the child experienced discomfort, prompting consultation. Physical examination revealed a soft, painless, compressible inguinoscrotal mass with bluish to reddish discoloration over the suprapubic and right inguinal areas.

**FIGURE 1:**
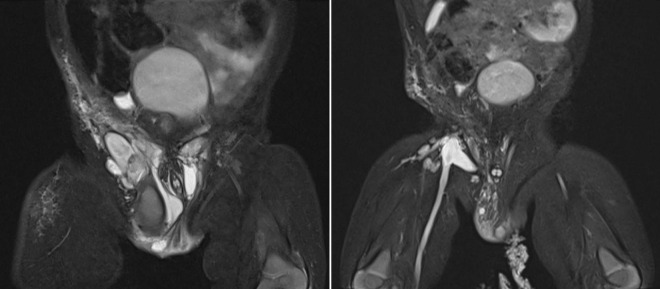
Pelvis MRI scans during the first treatment period (2020-2021). **Left image:** MRI scan before intervention showing multiloculated cystic masses with internal septations in the subcutaneous soft tissue of the mons pubis, extending to both groins, the right scrotum, and the lateral right thigh, with the largest cyst measuring 4 × 4 × 2 cm. **Right image:** MRI scan after the second sclerotherapy revealing a significant reduction in lesion size, with remaining cystic masses measuring 0.5–2 cm.

Doppler Ultrasound (US) and Magnetic Resonance Imaging (MRI) scan revealed multiloculated cystic masses with internal septation in the subcutaneous soft tissue of the mons pubis, extending to both groins, the right scrotum, and the lateral aspect of right thigh, with the largest cyst measuring 4 × 4 × 2 cm. The cystic fluid was hyperintense on T2-weighted images and hypointense on T1-weighted images, indicating intracystic hemorrhage. Post-contrast images showed septal enhancement, consistent with lymphatic malformation. Lamellar fluid-fluid levels indicated combined lymphatic and venous components of vascular malformations (LVMs), affecting the femoral vein and its tributaries (Figure 1). The left testis was in the scrotum, while the right testis was significantly smaller and located in the right inguinal canal.

Surgical excision and debulking of the LVMs, with right orchidopexy, were performed. Intraoperative findings revealed extensive infiltration of lymphatic and venous malformations into the suprapubic subcutaneous tissues, inguinal, scrotal, and right iliac fossa. The right testis was atrophic due to mass effect. Multiple cystic masses and dilated superficial veins were excised and sent for histopathology. Complete excision was not feasible due to the involvement of the iliac vessels and femoral vein. Histopathology showed thin-walled vascular channels lined by flattened endothelium, consistent with LVMs. No skin flap was necessary as only the severely discolored skin affected by LVMs was resected. The patient recovered well and was scheduled for monthly follow-up.

The patient only managed to return for check-up 6 months after the initial surgery with a recurrence of the lesion. MRI and US revealed a cystic mass in the right inguinal, scrotal, and pelvic regions, measuring 8 × 4 × 5 cm in its largest dimensions, with multiple signal voids on T2-weighted images suggesting phleboliths. Given the invasive nature of this LVM and its involvement with truncal vessels, complete excision was deemed impractical. Instead, sclerotherapy with intralesional Bleomycin injection, following the protocol, was indicated. Bleomycin 15 IU powder was diluted with NaCl 0.9% to a concentration of 1 IU/mL. Under general anesthesia, the cystic masses were percutaneously punctured and drained, and Bleomycin was injected at a maximum dosage of 0.5 IU/kg body weight. The solution was evenly distributed among the cysts, especially targeting those larger than 2 cm. Injections were also administered into the subcutaneous tissue of affected skin areas. The patient was discharged the next day and seen in the follow-up clinic after 2 months. At the 2-month follow-up, there was a reduction in lesion size and no discomfort. The skin had healed well, and the skin discoloration had resolved.

However, 6 months after the first Bleomycin dose, the lesion recurred and rapidly enlarged. MRI revealed a diffuse lesion in the scrotum and right inguinal canal, measuring 7 × 2 × 4 cm in its largest dimensions, with marked dilation of the right femoral vein and its branches, the right external iliac vein, and the right common iliac vein, without thrombosis. A second Bleomycin sclerotherapy was performed. Four months after the second Bleomycin injection, the patient showed a significant reduction in lesion size, with remaining cystic masses measuring between 0.5 and 2 cm (Figure 1). A summary of the first period of treatment (2020-2021) is provided in Table 1.

**TABLE 1:** Milestones of imaging studies and interventions during the first treatment period (2020-2021).

Timeline	Imaging study (MRI & Ultrasound)	Intervention
February 2020	A cystic mass with septations, the largest measuring 4 × 4 × 2 cm, was found along the inguinal canal and right scrotum, containing hemorrhagic fluid. Multiloculated masses extended to the mons pubis, groins, right scrotum, and thigh, with septal enhancement indicating LMs.	Surgical excision & debulking
August 2020	Imaging revealed an 8 × 4 × 5 cm cystic mass in the right inguinal, scrotal, and pelvic regions, with phleboliths and thick-walled cysts, indicating a lymphatic-venous malformation.	1^st^ Bleomycin injection
February 2021	A 7 × 2 × 4 cm diffuse lesion with heterogeneous enhancement was found in the scrotum and right inguinal canal, along with dilation of the right femoral, iliac veins, and superficial gluteal veins, without thrombosis.	2^nd^ Bleomycin injection
June 2021	Multiple cystic masses (0.5–2 cm) were found in the subcutaneous tissue of the right mons pubis, scrotum, groin, perineum, and thigh. The right femur appeared normal, and the dilated femoral, saphenous, and iliac veins had normal contrast uptake.	Watchful waiting

LMs, lymphatic malformations; LVMs, lymphatic-venous malformations; US, Doppler Ultrasound; MRI, Magnetic Resonance Imaging.

**FIGURE 2:**
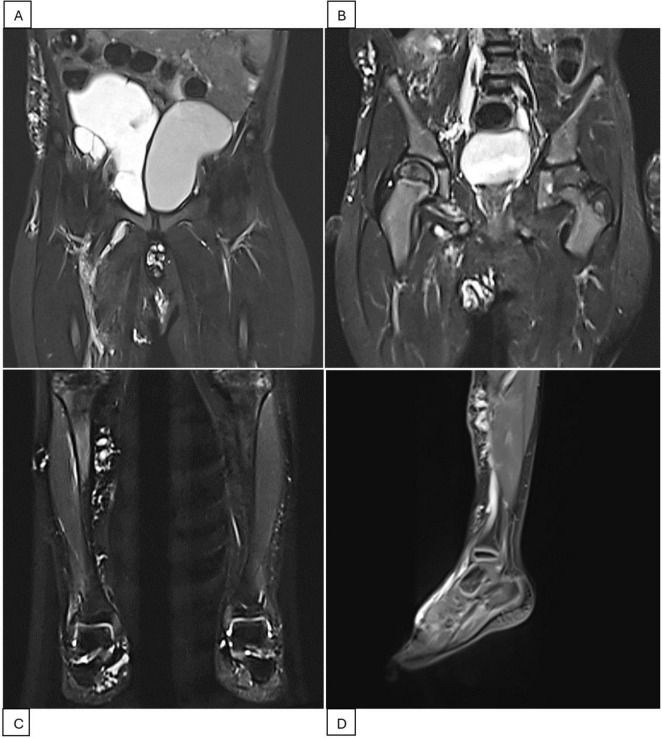
Pelvis and right leg MRI scans during the second treatment period (2023-2024). **(A)** MRI scan before intervention showing a cystic mass measuring 10 × 6 × 5 cm, displacing the bladder to the left. **(B)** MRI scan after the fourth Bleomycin injection session indicating resolution of the pelvic mass. **(C,D)** MRI scans of the right lower extremity showing scattered lesions in the right lower leg, ankle, and foot.

The patient was then lost to follow-up due to the COVID-19 outbreak and only returned 2 years later (2023). MRI showed diffuse infiltration of multiple cysts, with the largest being 0.4 cm, in the pelvic region, right iliac fossa, right groin, abdominal wall, suprapubic, and scrotal areas. Another large cystic mass was found in the right pelvic cavity, measuring 10 × 6 × 5 cm, and displaced the bladder to the left (Figure 2). The subcutaneous layer of the right iliac fossa was thickened and hyperechoic, with dilated venous structures. A fluid-filled mass of 4 × 1 cm with internal septations was present in the right iliac fossa. Scattered hyperechoic lesions were also noted in the right lower leg, ankle, and foot, with a few dilated veins. A third Bleomycin injection was subsequently performed.

Three months after the third Bleomycin session, the patient returned with significantly reduced cystic masses, with the largest lesion located in the pelvis measuring 5 × 3 cm. A fourth injection was administered. The patient is currently being closely monitored with follow-up visits every 2 months. During his last visit in April 2024, the LVMs were still under control. MRI showed LVMs in the abdominal wall, right inguinal-scrotal area, right buttock, and medial right thigh, measuring 1 × 1 cm. A venous aneurysm in the right inguinal region measured 1.6 × 1.5 cm without thrombosis, accompanied by adjacent lymphatic cysts in the right thigh. Scattered LVMs were also noted in the right lower leg, ankle, and foot (Figure 2). The patient experienced no symptoms, and the family had no concerns. Watchful waiting with regular 2-month follow-up was recommended. A summary of the second period of treatment (2023–2024) is provided in Table 2.

**TABLE 2:** Milestones of imaging studies and interventions during the second treatment period (2023-2024).

Timeline	Imaging study (MRI & ultrasound)	Intervention
September 2023	A 10 × 6 × 5 cm cystic mass in the right pelvis displaced the bladder, with smaller cysts in the pelvis, groin, and scrotal areas. The right iliac fossa showed thickened fat, dilated veins, and a 4 × 1 cm fluid-filled mass. Hyperechoic lesions and dilated veins were also present in the right lower leg and foot.	3^rd^ Bleomycin injection
December 2023	A cystic mass was located in the right pelvis, right lower anterior abdominal wall, and right inguinal-scrotal area. The largest cyst in the pelvis measured 5 × 3 cm and had internal septations	4^th^ Bleomycin injection
April 2024	LMs were present in the soft tissue of the abdominal wall, right inguinal-scrotal area, and right buttock. The subcutaneous tissue of the medial right thigh measured 1 × 1 cm. A venous aneurysm in the right inguinal region measured 1.6 × 1.5 cm, without thrombosis, accompanied by adjacent lymphatic cysts in the right thigh. Scattered LVMs in the right lower leg, ankle, and foot.	Watchful waiting

LMs, lymphatic malformations; LVMs, lymphatic-venous malformations; US, Doppler Ultrasound; MRI, Magnetic Resonance Imaging.

## Discussion

Misidentification and inconsistent naming of vascular anomalies are common across medical specialties, leading to an increased risk of improper management (2, 3). Historically, diagnosing and classifying vascular anomalies have been challenging due to their phenotypic diversity and evolving classification systems. To ensure effective communication and appropriate patient care, a standardized nomenclature is essential. The 2018 ISSVA system fulfills this need by offering the most up-to-date classification of vascular anomalies and is based on the widely accepted tumor/malformation dichotomy (3). In the ISSVA classification system, vascular anomalies are categorized into vascular tumors, which are neoplastic, and vascular malformations, which are non-neoplastic and result from structural anomalies of the vasculature. This distinction is based on the presence or absence of neoplastic proliferation of vascular endothelial cells (4).

The Hamburg classification system also plays a crucial role by categorizing vascular malformations based on anatomical and pathological characteristics. This system introduces the important distinction between “truncular” and “extratruncular” lesions. Truncular lesions involve larger, axial vessels, whereas extratruncular lesions affect smaller vessels within the tissue. These differences originate from the specific embryological stage at which development is disrupted (5). Although the ISSVA system did not initially include this distinction, the latest update incorporates truncular-type lesions. Advances in clinical, radiologic, histopathologic, and molecular knowledge have informed these updates, improving the understanding and management of vascular anomalies (2).

Lymphatic lesions with similar characteristics can manifest multifocally, affecting various tissues. These lesions are classified into two types based on their morphology: macrocystic lymphatic malformations (LM), previously known as cystic hygroma, and microcystic LM, previously known as lymphangioma (6). The distinction between these types is not always clear, and some patients may present with both types of lesions (6). LMs can coexist with other vascular anomalies, such as venous or capillary malformations, resulting in mixed vascular lesions (6, 7). Combined vascular malformations are defined as the presence of two or more distinct vascular malformations within a single lesion (4). According to the ISSVA classification, this case involves combined, multifocal lymphatic-venous malformations with both truncular and extratruncular components. The literature indicates that encountering such a complex case is very rare (2, 4, 6–8).

Ultrasound (US) and MRI are the primary imaging modalities for identifying, characterizing, and assessing the anatomic extent of vascular malformations and tumors (4, 9). While US is useful, MRI offers superior tissue characterization and allows for a more precise determination of lesion extension to critical structures like the orbit and spine (2). T2-weighted MRI is particularly effective in visualizing the extent of malformations, especially when lesions are located deep within the tissue (6, 7). In this patient, the combined use of US and MRI, with an emphasis on MRI, enabled early and accurate diagnosis and facilitated appropriate patient management.

An ideal treatment for lymphatic malformations (LMs) does not exist, and multiple interventions may be necessary (10). The lack of a standardized treatment algorithm for LMs is partly due to the heterogeneous presentation among patients (10). The primary treatment strategy should focus on addressing the specific symptoms present in each individual case. Curative treatment is not always feasible and should not be pursued aggressively, as it may lead to excessive and potentially harmful interventions (10). Indications for treating LMs include repeated infections within the malformation, recurrent intralesional hemorrhage, impaired mobility, functional impairment due to mass effect, aesthetic concerns, or other factors that significantly affect the quality of life (11).

Until recently, patients with lymphatic malformations (LMs) have been treated primarily with nonpharmacological approaches such as surgical resection, sclerotherapy, and laser therapy to provide local control and symptomatic relief (6, 9). However, these treatments are generally effective only for well-localized microcystic and macrocystic LMs (6). Macrocystic LMs typically respond well to sclerotherapy, whereas microcystic LMs are more challenging to treat with variable response rates (9, 11). Extensive microcystic LMs infiltrating normal soft tissue and bone may require extensive resections and local or free-flap reconstruction (10). When complete disease elimination is not feasible, multiple treatment modalities are combined to control the disease and achieve satisfactory functional outcomes (9, 10). Despite the treatment approach, there is always a risk of recurrence. Therefore, in many cases, LM treatment is symptomatic and necessitates lifelong therapy. Rarely, LMs may shrink and disappear spontaneously (7).

Bleomycin is a recognized sclerotic agent that induces a non-specific inflammatory response, resulting in thrombosis and fibrosis of cystic structures. It is also cost-effective and commonly used in oncology due to its accessibility (12). When comparing Bleomycin to other sclerotic agents for treating LMs, several advantages emerge. Doxycycline, for example, has reported complication rates ranging from 0 to 14%, including infections and hemorrhage (13). Lauromacrogol has complication rates up to 24%, may require more treatment sessions, and is less effective for larger or more complex LMs (14). Picibanil (OK-432), although generally well-tolerated, can cause significant systemic symptoms like fever, may be less effective for microcystic LMs, and its availability can be limited (13, 14).

Another agent that could be very useful for complex vascular malformations is sirolimus; however, it was not readily available at our institution. In clinical settings, it has emerged as a promising treatment for lymphatic anomalies (LAs) and other vascular disorders (15). Retrospective cohort studies have shown that 50–80% of LA patients treated with sirolimus experienced improvements in clinical symptoms, such as pain, bleeding, and functional deficits, as well as a reduction in lesion volume (15). Sirolimus also appears to be effective in reducing lesion size in intractable LMs (15).

While sclerotherapy for LMs is minimally invasive and often safe, it can still cause complications ranging from mild systemic symptoms like fever and fatigue to local swelling that compresses vital structures, potentially requiring prolonged intensive care (13). However, no adverse effects were observed in this patient.

Several limitations are present in this study, including the single-case design, lack of comparative data, potential bias, and restricted generalizability due to the condition’s rarity and the patient’s unique characteristics. As a result, further prospective studies comparing intralesional Bleomycin injection with alternative treatments are necessary.

## Conclusion

This case underscores the challenges in diagnosing and managing complex LVMs. It demonstrates the potential effectiveness of intralesional Bleomycin sclerotherapy as a treatment option, particularly when complete surgical excision is not feasible due to the involvement of critical structures. Despite the recurrence of the lesion and delays caused by the COVID-19 pandemic, repeated Bleomycin injections led to significant reduction and control of the LVMs, ultimately resulting in symptomatic relief and cosmetic improvement. Regular follow-ups confirmed the stability of the condition with no further complications or concerns. This case suggests that Bleomycin sclerotherapy can be a valuable tool in the management of LVMs, offering a minimally invasive alternative to more extensive surgical procedures.

## Data Availability

The datasets presented in this article are not readily available because they contain information that could compromise the privacy of research participants. Requests to access the datasets should be directed to Quang Thanh Nguyen, quang.nt@vinuni.edu.vn.
